# Indoor Environmental Quality in Mechanically Ventilated, Energy-Efficient Buildings *vs.* Conventional Buildings

**DOI:** 10.3390/ijerph121114132

**Published:** 2015-11-06

**Authors:** Peter Wallner, Ute Munoz, Peter Tappler, Anna Wanka, Michael Kundi, Janie F. Shelton, Hans-Peter Hutter

**Affiliations:** 1 Institute of Environmental Health, Center for Public Health, Medical University Vienna, Kinderspitalgasse 15, Vienna 1090, Austria; E-Mails: peter.wallner4@gmail.com (P.W.); michael.kundi@meduniwien.ac.at (M.K.); janie.shelton@gmail.com (J.F.S.); 2 Austrian Institute for Healthy and Ecological Building, Alserbachstraße 5, Vienna 1090, Austria; E-Mails: office@innenraumanalytik.at (U.M.); p.tappler@innenraumanalytik.at (P.T.); 3 Institute of Sociology, University Vienna, Austria, Alserbachstraße 5, Vienna 1090, Austria; E-Mail: anna.wanka@univie.ac.at

**Keywords:** energy-efficient buildings, indoor air quality, measurements, mechanical ventilation, natural ventilation

## Abstract

Energy-efficient buildings need mechanical ventilation. However, there are concerns that inadequate mechanical ventilation may lead to impaired indoor air quality. Using a semi-experimental field study, we investigated if exposure of occupants of two types of buildings (mechanical *vs.* natural ventilation) differs with regard to indoor air pollutants and climate factors. We investigated living and bedrooms in 123 buildings (62 highly energy-efficient and 61 conventional buildings) built in the years 2010 to 2012 in Austria (mainly Vienna and Lower Austria). Measurements of indoor parameters (climate, chemical pollutants and biological contaminants) were conducted twice. In total, more than 3000 measurements were performed. Almost all indoor air quality and room climate parameters showed significantly better results in mechanically ventilated homes compared to those relying on ventilation from open windows and/or doors. This study does not support the hypothesis that occupants in mechanically ventilated low energy houses are exposed to lower indoor air quality.

## 1. Introduction

By design energy-efficient homes (e.g., passive houses) need mechanical ventilation and heat recovery systems [1]. There is some evidence that built-in air ventilation systems in homes lead to an improvement in the subjectively assessed air quality and a reduction of reported health symptoms and ailments of the residents [2,3]. It can be assumed that the increased change of air associated with the mechanical ventilation systems in energy-efficient homes leads to an increased removal of pollutants, and thus to an overall improvement of the quality of indoor air [4,5]. On the other hand, there are concerns that the potential risks associated with these technical systems could nullify this advantage. The most frequently mentioned concerns are excess noise, increased draughts, concerns regarding the hygiene of the air duct system [6] and low humidity indoors due to an elevated volume of outdoor air in winter [7].

The aim of this study is to compare very energy-efficient resp. passive houses with controlled ventilation systems (including heat recovery systems) to conventional houses without mechanical ventilation. After 3 month of occupation we investigated indoor air quality, dust mite allergens, climatological factors, supply air flow and noise. After approximately one year, follow-up-measurements were performed.

## 2. Methods and Material 

New houses built according to very low energy or passive house standards (Austrian Standard B 8110-1) [8] with controlled ventilation systems with heat recovery systems formed the test group, whilst houses which corresponded to the normal building standards without mechanical ventilation systems formed the control group. In the buildings of the test group it was assumed that the air supply was provided both mechanically and via window ventilation whereas in the control group the fresh air supply was solely via ventilation through windows (and doors).

Participants and their residential properties were recruited with the help of institutions which have an overview of energy-efficient housing projects in Austria, articles in newspapers and newsletters and building companies. The investigated buildings were located in all provinces of Austria, the majority of them in Vienna and Lower Austria. All were built between 2010 and 2012. The first measurement date took place about three months (± three weeks) after the residents moved into the house, the follow-up appointment about one year (± three weeks) after the initial appointment.

The first measurements were performed between October 2010 and May 2012, the follow-up measurements between October 2011 and May 2013. No measurements were made during the warmer season (June to September). At both appointments measurements were made of indoor air pollutants (volatile organic compounds, aldehydes, mould spores, dust mite allergens, radon) and climatological factors of interior rooms (CO_2_ as ventilation parameter, temperature, humidity). Additionally, volumes of air supply and noise levels were measured in buildings with ventilation systems. In total, we investigated 62 homes with mechanical ventilation and 61 without mechanical ventilation (Table 1). In both groups, detached houses constituted approximately 70% of the sample, and apartments in multi-storey buildings the remaining 30%.

**Table 1:** Parameters measured in the investigated homes.

Parameter	Number of Homes Where Measurements Were Performed			
	TG M1	CG M1	TG M2	CG M2
VOC	61	61	61	59
Aldehydes	62	61	61	59
Mould spores	61	61	61	59
Dust mite allergens	62	60	57	56
CO_2_	62	61	61	59
Volume of supplied air	62	-	61	-
Radon (1 year)	62	60	-	-
Noise	14	-	-	-
Temperature	62	59	61	58
Relative humidity	62	60	61	59

TG = test group, CG = control group, M1 = 1^st^ measurement, M2 = follow-up-measurement.

The indoor air measurement planning and sampling strategies were based on the Standard series ISO 16000 and the specifications of the Austrian guideline for the evaluation of indoor air [9]. All measurements of indoor air pollutants were made under standardized conditions.

Volatile organic compounds (VOC) were sampled by pumping air through charcoal tubes (Anasorb 747, SKC, Eighty Four, PA, USA) according to the Austrian standard ÖNORM M 5700-2 [10]. Sampling was performed in the midst of living and sleeping rooms at a height between 1.2 and 1.5 m. Analysis was achieved using GC/MS (QP-2010S, Shimadzu, Kyoto, Japan). The calculation of the parameter “total VOC” was carried out by quantifying the total peak area using toluene as the calibration standard.

Sampling and analysis of aldehydes were achieved according to ISO 16000-2 [11] and ISO 16000-3 [12]. For sampling aldehydes DNPH (2,4-dinitrophenylhydrazine)-cartridges were used. Analysis was carried out by means of HPLC at the Austrian Federal Environment Agency.

Sampling of yeast and mould spores was carried out based on ISO 16000-16 [13], ISO 16000-19 [14] and VDI 4300 Part 10 [15]. For comparative purposes mould spores in outdoor air were also determined. An impaction air sampler (MAS-100, Merck, Darmstadt, Germany) was used; the nominal airflow rate was 100 liters per minute (±2.5%). As nutrient medium Dichloran-Glycerol (DG18) agar (Merck) was used. Culture media were incubated at 25 °C (±1 °C) for 3 to 7 days.

Dust was sampled from mattresses in sleeping rooms and from furniture with textiles (such as sofas) and rugs in living rooms with a vacuum cleaner. Dust mite allergens Der p1 and Der f1 were determined using enzyme-linked immunosorbent assay (ELISA). Analysis was performed at the BMA laboratory (Bochum, Germany).

Radon measurements were carried out in three rooms of each property according to ÖNORM S 5280-1 [16]. One year-track-etch-detectors (RSKS, Radosys Ltd., Budapest, Hungary) were used. The average annual radon concentration is calculated as the arithmetic mean of the individual concentrations measured in each of three rooms of each property.

The continuous determination (for one week) of the concentrations of CO_2_ in the bedrooms using a multifunction measuring instrument (Wöhler CDL 210, Mosway, Bad Wünnenberg) was based on VDI 4300 Part 9 [17]. Temperature and humidity were also measured with this instrument. As CO_2_ concentrations are strongly influenced by occupancy, we registered the number of persons and size of bedrooms where measurements took place.

Supply air flow was measured with two devices (a testovent 417, Testo, Lenzkirch, Germany and a FlowFinder-mk2, Retrotec Inc., Everson, WA, USA. The latter one is a zero pressure compensating device that uses an integrated fan to compensate for the resistance caused by the device.

The noise produced by the ventilation system was measured in all rooms for 30 s. A portable device (XL 2, NTi Audio AG, Schaan, Liechtenstein) was used. Analysis of the measurements was performed by the Spektrum-Center for Environmental Engineering and Management (Dornbirn, Austria).

## 3. Results and Discussion

### 3.1. Results

#### 3.1.1. Volatile Organic Compounds (VOC)

Total VOC (TVOC) concentrations in the properties (living rooms and bedrooms) with mechanical ventilation systems were, on both measurement dates, significantly lower (*p* < 0.01) than in the properties with only window ventilation (Table 2). 

**Table 2:** Indoor concentrations of total VOC, formaldehyde, sum of saturated acyclic aliphatic aldehydes and CO_2_.

	TG M1	CG M1	TG M2	CG M2
**TVOC**				
Median (µg/m^3^)	300	560	120	230
95th percentile	2100	4000	470	2500
>1000 µg/m^3^	19%	28%	1%	11%
**Formaldehyde**				
Median	27	40	22	31
95th percentile	53	67	46	59
>100 µg/m^3^	2%	1%	0%	0%
**Sum of saturated acyclic aliphatic aldehydes**				
Median	52	81	32	50
95th percentile	170	258	80	110
>100 µg/m^3^	19%	33%	2%	9%
**CO_2_**				
Median	1,360	1830	1280	1740
Maximum	3010	7190	2250	3780
>1000 ppm	84%	92%	89%	92%
>1400 ppm	45%	80%	33%	69%

TG = test group, CG = control group, M1 = 1st measurement, M2 = follow-up-measurement.

**Figure 1:**
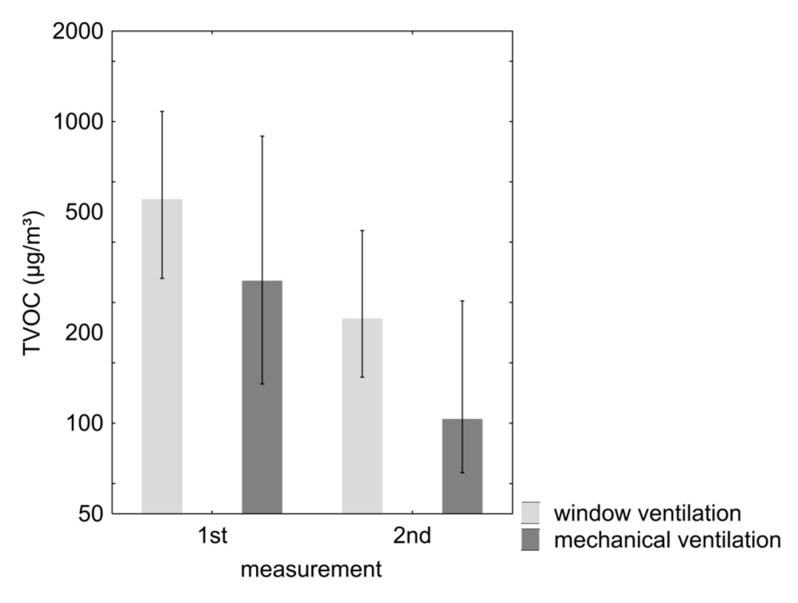
Median (and interquartile range) of TVOC at the 1st and 2nd measurement in mechanically and window ventilated properties (log scale).

The properties with mechanical ventilation had a median value at the first measurements of 300 µg/m^3^, while in the properties with window ventilation it was 560 µg/m^3^ (Figure 1). The TVOC concentrations decreased markedly in both property types in the period between the initial and the follow-up appointment. This change was statistically significant. However, an increase in TVOC values between the two measurement dates was seen in 17% of the studied rooms with mechanical ventilation and in 19% of the other rooms.

At the first appointment 3% of the rooms in properties with mechanical ventilation systems had TVOC values which were above 3000 µg/m^3^. In properties with only window ventilation 9% of these rooms were above this value. At the follow-up measurements there were no properties with mechanical ventilation systems which had a level above 3000 µg/m^3^, in properties with only window ventilation 3% of the rooms remained above this level.

#### 3.1.2. Aldehydes

**Figure 2:**
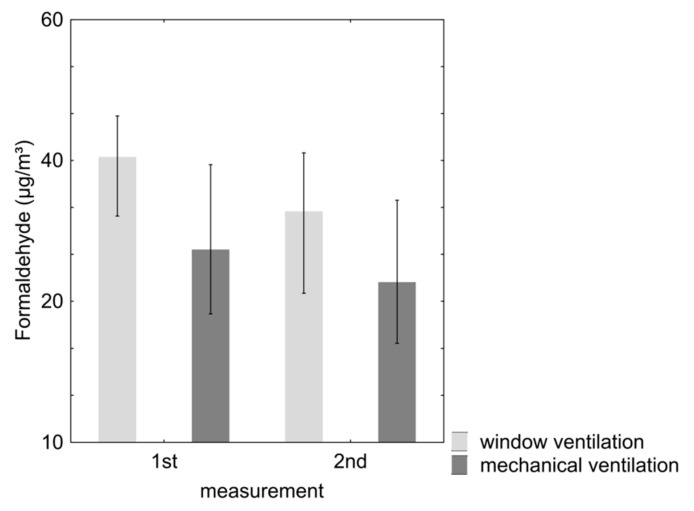
Median (and interquartile range) of formaldehyde at the 1st and 2nd measurement in mechanically and window ventilated properties (log scale).

The concentrations of formaldehyde in properties with mechanical ventilation were significantly lower (*p* < 0.001) than those in properties with only window ventilation (Table 2, Figure 2). The change in the concentration between the two measurement dates was significant for both types of houses. The group of properties with mechanical ventilation systems showed a reduction of the formaldehyde levels between the two measurement dates in 70% of the examined rooms, whereas an increase was noted in 23% of the rooms. Similarly, properties with only window ventilation showed a reduction in the concentration of formaldehyde in 80% of the cases, and 16% showed an increase.

Concentrations above the guideline level of 0.10 mg/m^3^ [18] were found in three rooms (first measurement). At the follow-up appointment, the value of 0.10 mg/m^3^ was not reached in any of the properties.

The pattern of reduction in the concentrations between the measurement dates was similar among other investigated aldehydes. In the lower aldehydes, acetaldehyde (median 1st measurement: 32 *vs.* 53 µg/m^3^; 2nd measurement: 18 *vs.* 33 µg/m^3^), maximum level 710 µg/m^3^) dominated in addition to formaldehyde. In the investigated higher aldehydes (saturated acyclic aliphatic C_4_–C_11_ aldehydes) the dominant substance was hexanal. At the first appointment, 33% of the values in the properties with only window ventilation exceeded the guideline of 100 µg/m^3^ for the sum of acyclic aliphatic C_4_–C_11_ aldehydes (Table 2).

#### 3.1.3. Mould Spores

At the first measurement, 84% of rooms in properties with mechanical ventilation systems had indoor concentrations (colony forming units CFU/m^3^) less than or equal to the concentrations in out-side air. In properties with only window ventilation, the percentage of rooms with a CFU concentration higher than outdoor air was 35%. In the follow-up measurements this was true for 21%, while only 10% of the properties with mechanical ventilation had a higher concentration indoors than outdoors.

#### 3.1.4. Dust Mite Allergens

At the first measurement allergen concentrations were slightly higher in properties with mechanical ventilation than window ventilation. Forty per cent of the properties with mechanical ventilation systems had values above 0.5 µg/g dust at the first appointment and 22% had a value above 2 µg/g (which is associated with a higher sensitization risk [19]). In properties with only window ventilation this was the case in 38% and 15% respectively. At follow-up measurements the percentage of values above 2 µg/g were almost identical (test group: 14%, control group: 12%). 26% of properties with mechanical ventilation systems had values above 0.5 µg/g dust and 32% of properties with only window ventilation. While the median was identical in both groups (<0.2 µg/g dust), the arithmetic mean was (insignificantly) higher in the control group.

#### 3.1.5. Carbon Dioxide

**Figure 3:**
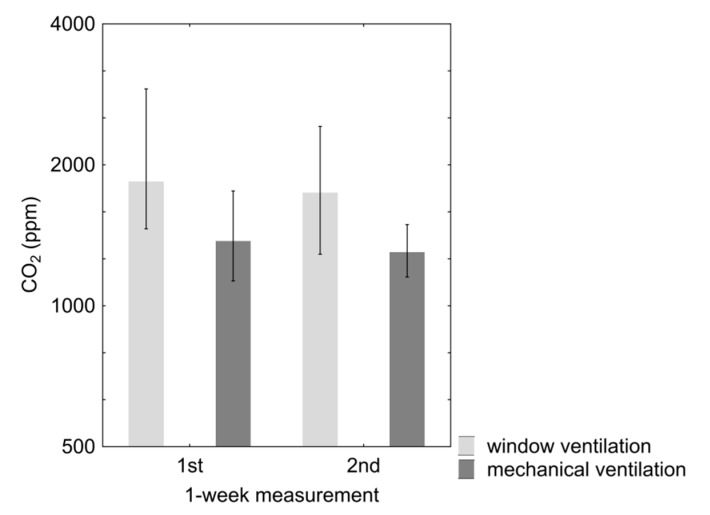
Median (and interquartile range) of maximum hourly average CO_2_ concentrations at the 1st and 2nd 1-week measurements in mechanically and window ventilated properties (log scale).

The median concentration of CO_2_ in bedrooms (Table 2) for properties with mechanical ventilation systems at the first appointment was 1360 ppm (1-week-measurement, maximum hourly average concentration) and for properties with only window ventilation it was 1830 ppm (follow-up measurements: 1280 *vs.* 1740 ppm). The difference was statistically significant (*p* < 0.01) (Figure 3).

The maximum hourly average CO_2_ concentration from both measurement dates for 75% of the properties with only window ventilation, and for 39% of the properties with mechanical ventilation systems were at least temporarily above the minimum hygienic standard of 1400 ppm (“low indoor air quality” according to EN 13779 [20]). Values above 1000 ppm (maximum hourly average concentration) were found in 84% and 89% of the test group (1st and 2nd measurement) and in 92% of the control group.

An analysis of the results for detached homes and apartments in multi-storey housing showed that with regard to CO_2_ concentrations in bedrooms there was no significant difference between the two residential types. During the first measurement, CO_2_ was measured in 16 properties with the ventilation systems both switched on and switched off. As would be expected, the concentrations were higher when the systems were switched off.

#### 3.1.6. Volume of Supplied Air

In approximately 85% of homes, the occupancy of the bedrooms was stated by the owners to be two or more people on both measurement dates for all properties with mechanical ventilation systems (in properties without such systems this investigation was not carried out). At the first appointment a supplied air flow rate in the bedrooms of at least 40 m^3^/h was reached in 7% of the properties, at the follow-up appointment, this number rose to 12%. The assessment of the volume of supplied air, taking into account the occupancy level, showed that at the first appointment only 11% of the bedrooms met the recommend level of supplied air of 20 to 25 m^3^/person*h (ÖNORM H 6038 [21]), by the follow-up visit this was 21%.

#### 3.1.7. Radon

The median of the radon concentrations (annual averages) in properties with mechanical ventilation systems was 17 Bq/m^3^, in properties with only window ventilation it was almost twice the concentration (31 Bq/m^3^) (*p* = 0.01). In one of the properties with only natural ventilation the value exceeded 200 Bq/m^3^, the Austrian guidance level for new buildings [22]. In three properties values were above 100 Bq/m^3^. In all properties with mechanical ventilation systems values were below 100 Bq/m^3^.

#### 3.1.8. Noise

The majority of the measurements made could not be evaluated because the normal operating noise of the ventilation system was below the background noise level. Thus the “signal” could not be identified. On the first measurement date the system noise level L_Aeq_, during normal operation conditions (level 2) was ≤ 23 dB in all cases (*n* = 14). At maximum ventilation level (level 3), 21% were > 25 dB (the guideline level of the Austrian Institute of Construction Engineering [23].

#### 3.1.9. Temperature and Humidity

The median relative humidity in the bedrooms of properties with mechanical ventilation systems at the first appointment was 40%, and 50% in those with window ventilation. At the second measurements the values were very similar (41% *vs.* 48%). In properties with mechanical ventilation systems it was slightly warmer than in properties with only window ventilation (median: 22 °C *vs.* 21 °C on both of the measurement dates). In summary, between the two measurements there were no significant changes in relative humidity or temperature in the bedrooms in either group.

### 3.2. Discussion

This investigation into the indoor air quality in highly energy-efficient buildings relative to conventional buildings was, to our knowledge the largest so far, with more than 3000 measurements of various pollutants and indoor parameters. The findings show that indoor air quality was higher in new highly energy-efficient buildings—which all had mechanical ventilation systems—than in new conventional properties. It is assumed, that this difference can be attributed to the increased flow of air in the properties with mechanical ventilation systems.

In many properties, especially with window ventilation, VOC values were elevated at the first measurement, as is often the case in new buildings [24]. This is due to VOC emissions from building materials and materials used for completion of the interior of the building. In 28% of the properties without mechanical ventilation TVOC values were above 1000 µg/m^3^ at the first measurement. Such concentrations are defined as “hygienically striking” by the German Committee on Indoor Guide Values [25]. According to the Austrian guideline on indoor air [9]), values over 1000 µg/m^3^ are regarded as “significantly raised”. After 12 months of residence, the levels in properties with mechanical ventilation systems were, with one exception, in an acceptable range below 1000 µg/m^3^, whereas in properties with only window ventilation the levels were still above 1000 µg/m^3^ in 11% of the rooms.

Levels above 3000 µg/m^3^ are described as “markedly elevated” in the Austrian guidelines; from the German Committee on Indoor Guidelines such concentrations are described as a “hygienic concern”; using such rooms is only acceptable for a short period of less than a month [25].

At the first appointment 3% of the living rooms and bedrooms in properties with mechanical ventilation systems had TVOC values which were above 3000 µg/m^3^. In properties with only window ventilation 9% of these rooms were also above this threshold. At the follow-up appointment there were no properties with mechanical ventilation which had a level above 3000 µg/m^3^, in properties with only window ventilation 3% remained above this level.

The TVOC medians in our study were 120 and 300 µg/m^3^, resp. in the rooms with mechanical ventilation, and 230 and 560 µg/ m^3^, resp. in those without.

There are numerous field studies of indoor VOC concentrations (e.g., [26,27,28,29,30]). The results of these studies are not always directly comparable due to different measurement methods and selection of the rooms measured. They do, however, provide relevant reference material in respect of the results of the current study.

As part of the Children’s Environmental Survey from 2003 to 2006 [26] 555 children’s rooms TVOC values were determined (using passive samplers), with a median of 300 µg/m^3^ and a 95-percentile value of 1000 µg/m^3^. Levels between 1000 and 3000 µg/m^3^ were measured in 3.4% of the households. TVOC concentrations above 3000 µg/m^3^ were not recorded in any children’s room.

Hutter *et al.* [28] investigated 160 randomly selected apartments in Vienna and found, by active sampling, total VOC concentrations in bedrooms with a median of 155 µg/m^3^. Of the examined apartments, 3% exceeded the value of 1000 µg/m^3^.

The AGÖF guidance values [31] for TVOC (median) is 360 µg/m^3^. The basis is found in a research project funded by the German Federal Environment Agency which included 4846 data sets of AGÖF institutions gathered during their investigations between 2006 and 2012.

The total VOC values in the studied properties at their follow-up appointment were generally in the range as found in the study which was carried out about 10 years ago by Hutter *et al.* [28] and in the range of AGÖF guidance values. VOC concentrations were highly significantly lower in the properties with mechanical ventilation systems, but were still raised in 19% of the properties (first measurements). This shows that the mechanical ventilation systems rendered a significant improvement in comparison with conventional buildings, although these could not be regarded as sufficient when they were the sole measure. There is a potential for a large reduction in VOC emissions if less emitting materials are used (and use is supervised).

A study by Coutalides *et al.* [32] showed significant differences between quality-assured and not quality-assured buildings, in terms of total VOC concentrations in properties in which measurements were made between 30 and 100 days after building completion, albeit before anyone moved in and before the installation of furniture. In properties where building was carried out with construction supervision, a median value TVOC of 480 µg/m^3^ was found, for properties without construction supervision it was 1100 µg/m^3^.

Regarding aldehydes, according to the WHO definition, formaldehyde, the simplest aldehyde, actually belongs to the very volatile organic compounds group [33] and is usually treated separately because of its importance in indoor air quality. Other very volatile aldehydes, such as acetaldehyde, have a less important role.

Higher aldehydes have a special position among the volatile compounds occurring indoors, as there is generally no primary source for those substances; they arise mainly in the room itself as products of reactions between substances found in building materials and in materials used for finishing the interior of the building. Examples of these are the formation of higher aldehydes from alkyd resin paints or products which contain linseed oil, such as oil-containing impregnations or linoleum [34]. The most common higher aldehyde found indoors is hexanal.

The investigations show that the measured concentrations of formaldehyde, with few exceptions, were of a rather low level. Properties with mechanical ventilation systems had highly significant lower values in the bedrooms and living rooms where measurements were taken, on both of the measurement dates when compared with the properties with only window ventilation. As is the case with VOC, it is assumed that this difference is due to the continuous increased air supply in properties with living room ventilation systems.

The guideline value of 0.10 mg/m^3^ [18] for formaldehyde was only exceeded in a few of the examined rooms (*n* = 3) on the first measurement date. At the follow-up appointment, the value of 0.10 mg/m^3^ was not reached in any of the properties. Median levels of formaldehyde were between 22 and 40 µg/m^3^.

As for VOC, there are numerous field investigations regarding formaldehyde concentrations in indoor rooms. A larger number of older studies reported formaldehyde concentrations up to several milligrams per cubic meter in non-commercial indoor rooms [35]. An older study of 100 Austrian apartments showed that in 97% of investigated cases, formaldehyde concentrations above 0.05 ppm (0.06 mg/m^3^) were found, and in 79% of cases above 0.1 ppm (0.12 mg/ m^3^) [36].

Krause *et al.* conducted a study in Germany where a total of 329 randomly selected apartments were investigated by means of passive samplers of formaldehyde [37]. The median value was 0.044 ppm (≈53 µg/m^3^); the highest measured value was 0.247 ppm.

Hutter *et al.* [28] recorded values between 0.007 and 0.092 ppm (about 8 and 100 µg/m^3^) via active sampling of formaldehyde concentrations in 160 bedrooms of randomly selected apartments in Vienna. 6 apartments had values above 0.05 ppm (60 µg/m^3^), the median was 0.02 ppm (24 µg/m^3^). In the KUS study [26] almost the same median was found (23.5 µg/m^3^). AGÖF gives a guidance value for the formaldehyde median of 35 µg/m^3^ [31].

Compared to earlier Austrian and German studies it can be seen that the concentrations of formaldehyde are markedly lower than those from about 20–25 years ago. The values found at the follow-up appointment in the examined properties were of a similar order of magnitude to those found in Vienna by Hutter *et al.* [28]. The results showed further that the efforts of the legislature and the wood products industry in Austria since the mid-1980s have been successful.

The AGÖF guidance level (median) for acetaldehyde is 20 µg/m^3^. In the KUS study [26] the acetaldehyde median was 15.5 µg/m^3^. The median in our study was between 18 and 53 µg/m^3^, with the lower levels found in the buildings with mechanical ventilation. The guideline level of the German committee on Indoor Guide values (100 µg/m^3^) [38] was only exceeded in rooms with only window ventilation. There was a decrease in the concentrations at the second measurement.

The concentration of higher aldehydes (sum of acyclic aliphatic C_4_–C_11_ aldehydes) at the first measurements was above the German guideline level value of 100 µg/m^3^ [39] in a considerable percentage. At the follow-up appointment, only 2% of the values in the properties with mechanical ventilation systems were above this value, and 9% of the values in the properties with only window ventilation.

For a more differentiated assessment of mould concentrations, genera and species had to be considered. Nevertheless, from a purely quantitative perspective, deductions can be made based on the differences between indoor and outdoor air. The background concentration of mould spores indoors is affected by the highly variable concentration of spores outdoors, which in turn depends on their local environment and their vegetation period. The concentration of mould spores indoors cannot therefore be considered in isolation from the outdoor concentration [40].

Elevated concentrations of spores in the indoor air can be caused when activities in the room are such, that they cause a resuspension of dust as this may contain increased numbers of sedimented spores. It is also possible that outdoor air, which has usually higher numbers of spores, leads to a pollution of the indoor air, while there is no primary source of mould in the room itself. If there are more CFU per m^3^ detected in the indoor air than outside this is an indication for a possible mould source indoors. The indoor/outdoor ratio and difference is often used in studies and for evaluations [40,41,42].

This study showed that the house type (with or without mechanical ventilation system) had a significant influence on the mean difference of the concentration of colony forming units between outside air and indoor air (negative values indicate a possible indoor mould source). When comparing the different house types, fewer properties with mechanical ventilation systems had indications of an indoor source of mould than properties with only window ventilation, at both measurement dates.

The results of the dust mite allergen measurements are likely to have a direct correlation with the origin of the furniture from which samples were taken. At the first measurement date, the results were probably dependent on whether pieces of furniture such as sofas, carpets and mattresses had been brought from the previous residence or were newly acquired. For this reason a differentiation between the two house types with regard to dust mites at the first appointment is not meaningful, since this time point was too early to provide information about the influence of the type of house. An explanation for the higher values in the group of properties with mechanical room ventilation systems at the first appointment could not be found.

At the second measurement date it can be assumed that in the majority of cases an accumulation of allergens over a one year period had taken place (although it is not known whether and to what extent new purchases or replacement of furniture had taken place between the two measurement dates). At the follow-up appointment, the arithmetic mean of the concentrations in the properties with mechanical ventilation systems was lower than that in the properties with only window ventilation. However, the differences between the types of houses were not significant.

The concentration of CO_2_ indoors is mainly used as a general indicator of the total amount of organic emissions and odorous substances emitted by people. It can be considered as an indicator of the level of ventilation with outdoor air. For evaluation purposes, both the classification of the EN 13779 [20] and the CO_2_ guideline of the indoor air working group of the Austrian environmental ministry [9] have been used.

As expected, the CO_2_ concentrations in bedrooms of properties with mechanical ventilation were at both measurement dates highly significantly lower than those measured in the properties with only window ventilation. Nevertheless, in the bedrooms with mechanical ventilation systems, at the first appointment, 84% of the rooms had maximum hourly average values of >1000 ppm indicating that they were at least temporarily at a level of moderate or even lower indoor air quality according to EN 13779 (89% at the follow-up appointment). 

This lack of air supply in the bedrooms of the properties with mechanical ventilation systems was also reflected in supplied volume of air as calculated per person. The hygienically desirable external air supply per person in bedrooms, according to ÖNORM H 6038 (2014) [21], is 20 to 25 m^3^/person m^3^/person*h. Taking into consideration the occupancy level at the first appointment only 11% of the bedrooms conformed to this standard range, at the follow-up measurements this was 21%.

The reason for the unexpectedly low volume of air supply in the bedrooms of the properties and the surprisingly high CO_2_ concentrations was most likely due to the specifications in the outdated version of the Austrian Standard H 6038 (2006), the standard was still valid at the time of the investigation. The standard gave a requirement for the general change of air in relation to the entire volume of the property, but describes no particular requirements in respect of the volume of air supply per person for critical areas such as bedrooms.

The yearly average value for radon was markedly lower in objects with mechanical ventilation systems (17 *vs.* 31 Bq/m³). This was probably due to the structurally related air-tight design of the building as a whole and the higher level of air exchange.

With regard to noise of the ventilation systems Austrian building regulations [23] as well as the “comfort ventilation” standards [43] were met in all of the assessed properties during normal operating conditions.

An interpretation of the values for the relative humidity can only be made with caution as humidity was only measured for one week. Relative humidity was significantly lower in the bedrooms with mechanical ventilation. Because of the higher air exchange in the properties with mechanical ventilation systems during the colder seasons of the year a greater amount of dry outside air gets into the building. Thus a generally lower humidity can be expected. This in turn can lead to complaints that the air is too dry. Low values below 30% were found almost exclusively in properties with mechanical ventilation systems, values above 55% (mould growth risk) were almost exclusively found in rooms with only window ventilation. The problem of low relative humidity in energy-efficient houses should be tackled with moisture recovery and other strategies**.**

A strength of this study is the high number of measurements (>3000) made in more than 120 homes. A weakness is the fact that for organisational reasons in many cases the measurement intervals were rather low or only spot samplings were possible.

## 4. Conclusions

To our knowledge this investigation is the largest study so far on this issue. Both types of houses investigated (highly energy-efficient with mechanical ventilation *vs.* conventional) were built at almost the same time.

This study shows that indoor air quality in energy-efficient new houses (private homes, with mechanical ventilation) was higher than in conventional new buildings. This was true for almost all investigated parameters like, inter alia, TVOC, aldehydes, CO_2,_ radon, and mould spores.

It would be interesting to investigate the mechanically ventilated properties in, e.g., 5 years again to see if maintenance regimes concerning the air ducts have an influence on indoor air quality.
